# High‐flow nasal cannula oxygen therapy for the treatment of acute respiratory failure secondary to SARS‐CoV‐2 pneumonia out of ICU

**DOI:** 10.1111/crj.13679

**Published:** 2023-08-04

**Authors:** Sonia Castro, Sandra Pedrero, Luis Alberto Ruiz, Leyre Serrano, Rafael Zalacain, Silvia Pérez‐Fernández, Milagros Iriberri, Valentín Cabriada

**Affiliations:** ^1^ Pneumology Service Hospital Universitario de Cruces Barakaldo Bizkaia Spain; ^2^ Department of Medicine and Surgery, Facultad de Medicina y Enfermería Universidad del País Vasco/Euskal Herriko Unibertsitatea UPV/EHU Leioa Bizkaia Spain; ^3^ Department of Immunology, Microbiology and Parasitology, Facultad de Medicina y Enfermería Universidad del País Vasco/Euskal Herriko Unibertsitatea UPV/EHU Leioa Bizkaia Spain; ^4^ Scientific Coordination Facility Biocruces Bizkaia Health Research Institute Barakaldo Spain

**Keywords:** COVID‐19, non‐invasive ventilation, oxygen inhalation therapy, respiratory insufficiency, respiratory therapy

## Abstract

**Introduction and objectives:**

High‐flow nasal cannula oxygen therapy (HFNC) has been successfully used for the treatment of acute hypoxaemic respiratory failure (AHRF) secondary to SARS‐CoV‐2 pneumonia and being effective in reducing progression to invasive mechanical ventilation. The objective of this study was to assess the usefulness of HFNC on a hospital ward for the treatment of AHRF secondary to SARS‐CoV‐2 pneumonia and its impact on the need for intensive care unit (ICU) admission and endotracheal intubation. Other objectives include identifying potential physiological parameters and/or biomarkers for predicting treatment failure and assessing the clinical course and survival.

**Methods:**

Observational study based on data collected prospectively between March 2020 and February 2021 in a single hospital on patients diagnosed with AHRF secondary to SARS‐CoV‐2 pneumonia who received HFNC outside an ICU.

**Results:**

One hundred and seventy‐one patients out of 1090 patients hospitalised for SARS‐CoV‐2 infection. HFNC was set as the ceiling of treatment in 44 cases; 12 survived (27.3%). Among the other 127 patients, intubation was performed in 25.9% of cases with a mortality of 11.8%. Higher creatinine levels (OR 1.942, 95% CI 1.04; 3.732; *p* = 0.036) and Comorbidity‐Age‐Lymphocyte‐LDH (CALL) score (OR 1.273, 95% CI 1.033; 1.617; *p* = 0.033) were associated with a higher risk of intubation. High platelet count at HFNC initiation was predictive of good treatment response (OR 0.935, 95% CI 0.884; 0.983; *p* = 0.012).

**Conclusions:**

HFNC outside an ICU is a treatment with high success rate in patients with AHRF secondary to SARS‐CoV‐2 pneumonia, including in patients in whom this therapy was deemed to be the ceiling of treatment.

## Graphical abstract

High‐flow nasal cannula is a treatment with high success rate for patients with acute respiratory failure secondary to SARS‐CoV‐2 pneumonia. This therapy reduces the rates of intubation and mechanical ventilation. It can be used effectively in a conventional hospital ward. In patients not candidates for critical care may also be a suitable treatment option.

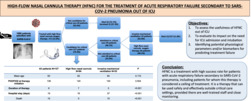

## 1 INTRODUCTION

Acute hypoxaemic respiratory failure (AHRF) is a common and severe complication of patients with SARS‐CoV‐2 pneumonia. Treatment of this condition often requires admission to an intensive care unit (ICU), and in many cases, prolonged invasive mechanical ventilation (IMV). In a study in Italy, 88% of 1591 patients admitted to intensive care units for SARS‐CoV‐2 required IMV.^1^ In Spain, an analysis of data from the SEMI‐COVID registry on 15 111 patients hospitalised for SARS‐CoV‐2 found that 33.1% of patients developed acute respiratory distress syndrome and 79.5% of those admitted to an ICU required IMV, with an overall mortality rate of 21%.^2^ In other multicentre study of 663 patients admitted to ICUs, 74% required IMV, and the mortality rate was 31%.^3^

The large number of patients infected with this virus overwhelmed ICUs and non‐invasive respiratory support (NIRS) started to be used outside this setting. One of the most widely used systems has been high‐flow nasal cannula oxygen therapy (HFNC), which has been successfully used in the treatment of acute respiratory failure due to other causes and has shown to be effective in reducing progression to IMV in the case of SARS‐CoV‐2 infection.^4^, ^5^, ^6^ Although numerous studies have been reported on the use of NIRS and specifically HFNC in patients with SARS‐CoV‐2 infection, most of these have been carried out in ICUs. Few studies have been conducted in patients who have received this therapy on the hospital ward.

Our hypothesis was that the use of HFNC is feasible outside critical care units, improves the course of the disease and reduces the need for ICU admission and intubation. If our hypothesis is confirmed, this would represent a breakthrough in the treatment of patients with AHRF.

The main objective of this study was to assess the usefulness of HFNC in a conventional hospital ward for the treatment of severe AHRF secondary to SARS‐CoV‐2 pneumonia and its impact on the need for ICU admission and intubation. Other objectives included identifying potential physiological parameters and/or biomarkers for predicting treatment failure and assessing the clinical course and survival of this cohort of patients.

## 2 METHODS

### 2.1 Study population and design

Observational study based on the analysis of a prospective registry of all patients diagnosed with SARS‐CoV‐2 pneumonia admitted to a hospital ward of the Pneumology Service at Cruces University Hospital (Barakaldo, Spain) between March 2020 and February 2021. Patients included in the analysis were ≥18 years old and had PaO2/FiO2 ≤ 200 mmHg. We selected patients with AHRF treated with HFNC. The diagnosis of SARS‐CoV‐2 infection was based on a positive result in a polymerase chain reaction or antigen test of a nasopharyngeal swab sample. Patients were excluded from the study if they presented immediate need for orotracheal intubation.

We collected data on patient demographic characteristics, comorbidities, clinical, radiological and laboratory findings. We calculated the Comorbidity‐Age‐Lymphocyte count‐Lactate dehydrogenase (CALL) score, considering scores <7 indicative of a low risk of progression.^7^ We considered the following variables as indicators of clinical progression: (1) ICU admission; (2) use of IMV; (3) length of hospital stay; and (4) all‐cause mortality. Regarding variables associated with the respiratory therapy under study, we collected data on (1) time course of the disease (from the start of the symptoms until admission to hospital and initiation of HFNC); (2) arterial oxygen partial pressure‐to‐fractional inspired oxygen ratio (PaO2/FiO2) at the start of the therapy; and (3) duration of HFNC.

As routine practice, our hospital held a daily clinical meeting between ICU staff and physicians responsible for patients with SARS‐CoV‐2 infection on hospital wards. In these meetings, we decided the approach to be taken in the event of worsening in relation to ICU admission and intubation for patients with severe acute respiratory failure secondary to this viral infection. The following were taken into account: the presence and severity of comorbidities, frailty and likelihood of a good outcome after intubation. For the management of patients on the conventional ward, the Pneumology Service drafted a treatment protocol in which the use of HFNC was prioritised over other types of NIRS given that it is easy to use and well tolerated by patients. Treatment with this type of therapy was considered if PaO2/FiO2 fell to below 200 mmHg.

### 2.2 Respiratory therapy

We used OH‐60A (Micomme) and Airvo 2 (Fisher & Paykel Healthcare) devices. Treatment was started with high flows between 60 and 70 L/min depending on patient tolerance, at a temperature between 31 and 37°C, adjusting Fi02 to maintain oxygen saturation (SatO2) > 94%. Patients were treated on a conventional hospital ward additionally equipped to enable non‐invasive monitoring of oxygen saturation and heart rate. All patients wore surgical masks during the therapy to reduce the spread of the virus. HFNC was withdrawn, on a case‐by‐case basis depending on the clinical course of the patient, progressively reducing the oxygen flow and FiO2 to 25–30 L/min and 30%, respectively. When deemed necessary, the decision concerning the point at which it was appropriate to perform intubation and start IMV was made by the medical team based on the patient's clinical status and SatO2 in relation to the FiO2 administered. HFNC was considered to have failed in patients who required intubation and/or died.

### 2.3 Statistical analysis

Normally and non‐normally distributed quantitative variables were expressed as mean (and standard deviation) and median (and interquartile range), respectively. To identify differences between groups we used, Student's *t*‐test if data were normally distributed, and otherwise, the non‐parametric Mann–Whitney *U* test. Qualitative variables were expressed as percentages and compared using chi‐square or Fisher's exact tests. Multivariate analysis was performed to assess variables associated with HFNC failure using logistic regression models. All variables that were found to be significant in the univariate analysis and had clinical significance were included in these models. The analysis was carried out using the R statistical software.

## 3 RESULTS

**FIGURE 1:**
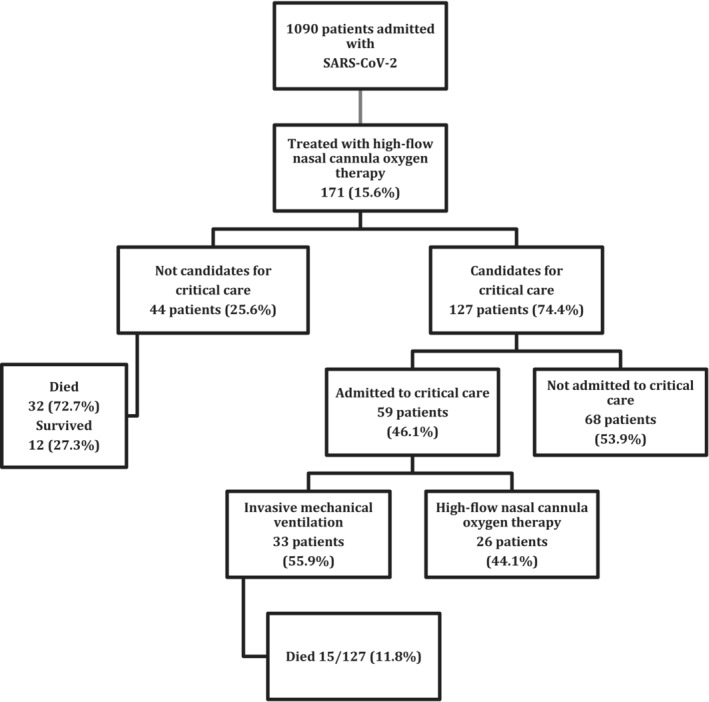
Flow of patients through the study.

During the study period, out of 1090 patients admitted to our hospital for SARS‐CoV‐2, a total of 171 (15.6%) were treated with HFNC on the ward (Figure 1). Treatment with HFNC was successful in 106 out of these 171 patients (61.9%).

The general characteristics of the group and the details concerning HFNC are summarised in Table 1. The median age was 66 years (IQR 58–75); 70.8% of the patients were male. The main comorbidities observed were hypertension (43.9%), dyslipidaemia (42.1%) and diabetes mellitus (25.7%). Patients received pharmacological treatment in accordance with the guidelines prevailing at the time. A total of 41 patients (27.5%) died.

**TABLE 1:** Demographic and clinical characteristics of patients given high‐flow nasal cannula oxygen therapy and details concerning with respiratory therapy.

	All patients *N* = 171
Demographic characteristics	
Sex (male)	121(70.8%)
Median age	66.0 [58.0;75.0]
Median body mass index (kg/m^2^)	28.1 [25.1;31.2]
Active smoking	9 (5.26%)
Alcohol abuse	25 (14.6%)
Comorbidities	
Hypertension	75 (43.9%)
Dyslipidaemia	72 (42.1%)
Diabetes mellitus	44 (25.7%)
Heart disease	26 (15.2%)
Lung disease	32 (18.7%)
Kidney disease	21 (12.3%)
Liver disease	15 (8.77%)
Clinical characteristics assessed in the emergency department	
Systolic blood pressure (mmHg)	128 [115;142]
Diastolic blood pressure (mmHg)	74.7 (11.6)
Body temperature (°C)	37.1 (0.92)
Respiratory rate (rpm)	16.0 [16.0;22.0]
Heart rate (lpm)	90.0 [80.0;101]
Mean oxygen saturation (%)	93.5 [90.0;96.0]
Blood test results	
Creatinine (mg/dL)	0.84 [0.69;1.07]
C‐reactive protein (mg/L)	98.1 [55.5;155]
Procalcitonin (ng/mL)	0.11 [0.06;0.24]
Lactate dehydrogenase (U/L)	375 [307;461]
Ferritin (ng/mL)	995 [526;1664]
Hospital stay (days)	17 [12.0;27.0]
CALL score	10 [8.00;12.0]
Death	47 (27.5%)
Treatment duration (days)	7.00 [4.00;10.5]
Ratio PO2/FiO2	128 [90.0;154]
<100	52 (30.4%)
≥100	119 (69.6%)
Time to initiation of therapy from admission (days)	3.00 [1.00;5.00]
Time to initiation of therapy from onset of symptoms (days)	10.0 [7.00;12.0]

*Note*: Data are expressed as mean (SD), median and interquartile range (IQR) and *N* (%).

The multidisciplinary team considered that 44 patients (25.6%) were not candidates for invasive procedures. In this group, the mean age was 80 years old (76–84), and 72.7% of the patients were male. Hypertension was the most common comorbidity, and the mean PaO2/FiO2 at the start of the therapy was 84. A total of 12 of these 44 patients (27.3%) survived.

The other patients (*n* = 127, 74.4%) were considered candidates for invasive procedures. Table 2 summarises the baseline characteristics of this cohort, overall and stratified by intubation. The patients not intubated had lower CALL scores, lower levels of creatinine, lactate dehydrogenase and procalcitonin and a higher platelet count. A total of 68 patients (53.9%) progressed well with the HFNC given on the hospital ward, but 59 patients (46.1%) were admitted to the ICU. Of the latter group, 26 remained on HFNC, and the other 33 were intubated, 15 of them dying. In the multivariate analysis, a higher creatinine level (OR 1.942, 95% CI 1.04; 3.732; *p* = 0.036) and CALL score (OR 1.273, 95% CI 1.033;1.617; *p* = 0.033) were independently associated with a higher risk of intubation. In contrast, a higher platelet count at the start of HFNC was found to be predictive of a good response to this therapy (OR 0.935, 95% CI 0.884; 0.983; *p* = 0.012).

**TABLE 2:** Characteristics of patients who were candidates for invasive procedures.

	All patients *N* = 127	Patients treated with high‐flow nasal cannula oxygen therapy *N* = 94	Patients treated with invasive mechanical ventilation *N* = 33	*P*
Demographic characteristics				
Sex (male)	89 (70.1%)	66 (70.2%)	23 (69.7%)	1.000
Mean age	63.0 [55.0;70.0]	62.0 [52.0;69.8]	64.0 [59.0;70.0]	0.172
Mean body mass index (kg/m^2^)	27.9 [25.0;31.1]	27.5 [25.0;30.9]	28.3 [25.7;31.7]	0.415
Active smoking	6 (4.72%)	3 (3.19%)	3 (9.09%)	0.424
Alcohol abuse	20 (15.7%)	13 (13.8%)	7 (21.2%)	0.110
Comorbidities				
Hypertension	48 (37.8%)	31 (33%)	17 (51.5%)	0.093
Dyslipidaemia	51 (40.2%)	38 (40.4%)	13 (39.4%)	1.000
Diabetes mellitus	26 (20.5%)	18 (19.1%)	8 (24.2%)	0.709
Heart disease	10 (7.87%)	8 (8.51%)	2 (6.06%)	1.000
Lung disease	22 (17.3%)	16 (17%)	6 (18.2%)	1.000
Kidney disease	10 (7.87%)	5 (5.32%)	5 (15.2%)	0.125
Liver disease	9 (7.09%)	5 (5.32%)	4 (12.1%)	0.237
Usual treatment				
ACE inhibitors/ARBs	35 (27.6%)	21 (22.3%)	14 (42.4%)	0.046
Statins	39 (30.7%)	28 (29.8%)	11 (33.3%)	0.872
Anticoagulants	11 (8.66%)	8 (8.51%)	3 (9.09%)	1.000
Platelet aggregation inhibitors	18 (14.2%)	13 (13.8%)	5 (15.2%)	1.000
Oral corticosteroids	6 (4.72%)	1 (1.06%)	5 (15.2%)	0.005
Clinical characteristics assessed in the emergency department				
Systolic blood pressure (mmHg)	125 [115;142]	128 [116;144]	120 [110;137]	0.092
Diastolic blood pressure (mmHg)	75.4 (11.3)	76.1 (11.4)	73.3 (11.0)	0.230
Body temperature (°C)	37.1 (0.91)	37.1 (0.93)	37.2 (0.85)	0.490
Respiratory rate (rpm)	16.0 [16.0;22.0]	16.0 [16.0;22.0]	16.0 [15.0;22.0]	0.932
Heart rate (lpm)	93.5 (15.9)	92.6 (14.9)	96.3 (18.2)	0.292
Mean oxygen saturation (%)	94.0 [91.0;96.0]	94.0 [91.0;96.0]	94.0 [91.0;96.0]	0.726
Blood test results				
Creatinine (mg/dL)				
In the ED	0.91 [0.76;1.10]	0.90 [0.74;1.06]	1.04 [0.88;1.39]	0.003
At therapy initiation	0.81 [0.67;0.97]	0.78 [0.65;0.92]	0.95 [0.74;1.24]	0.003
C‐reactive protein (mg/L)				
In the ED	95.2 [52.7;154]	93.2 [45.2;157]	101 [73.8;149]	0.545
At therapy initiation	92.5 [49.8;150]	91.2 [46.4;151]	94.1 [56.0;149]	0.733
Procalcitonin (ng/mL)				
In the ED	0.13[0.06;0.32]	0.12[0.05;0.23]	0.20[0.09;0,49]	0.121
At therapy initiation	0.11 [0.06;0.18]	0.09 [0.05;0.16]	0.20 [0.10;0.49]	0.004
Lactate dehydrogenase (U/L)				
In the ED	342 [282;411]	341 [272;392]	361 [299;425]	0.384
At therapy initiation	361 [304;442]	356 [297;422]	419 [329;476]	0.008
Ferritin (ng/mL)				
In the ED	830 [404;1466]	828 [372;1492]	831 [457;1195]	0.690
At therapy initiation	1036 [563;1739]	1021 [550;1713]	1086 [696;1730]	0.601
Lymphocytes/μL				
In the ED	810 [650;1058]	830 [660;1080]	790 [630;1010]	0.681
At therapy initiation	725 [542;955]	760 [560;980]	650 [520;890]	0.176
Platelets ×10^3^/μL				
In the ED	171 [135;221]	175[136;233]	156 [131;200]	0.085
At therapy initiation	216 [168;302]	244 [176;315]	187 [155;216]	0.001
D‐dimer (ng/mL)				
In the ED	620 [418;965]	600 [370;952]	675 [532;1025]	0.315
At therapy initiation	630 [385;1215]	600 [380;1085]	720 [430;1255]	0.814
PO2/FIO2 at therapy initiation	138 [103;160]	140 [102;165]	130 [108;148]	0.504
Duration of therapy	6.00 [4.00;10.0]	7.00 [5.00;11.0]	3.00 [1.00;5.00]	<0.001
CALL score	10 [7.0;12.0]	8.50[6.0;12.0]	10[8.0;12.0]	0.037
Low CALL score (<7)	22 (21,8%)	21 (27,6%)	1 (4%)	0.028
High CALL score (≥7)	79 (78,2%)	55 (72,4%)	24 (96%)	
Hospital stay (days)	19 [13.0;29.0]	16[12.0;21.8]	37 [27.0;52.0]	<0.001
Death	15 (11.8%)	0 (0.00%)	15 (45.5%)	<0.001

*Notes*: Data are expressed as mean (SD), median and interquartile range (IQR) and *N* (%). ‘Therapy’ refers to high‐flow nasal cannula oxygen therapy.

Abbreviations: ACE, angiotensin‐converting enzyme; ARBs, angiotensin II receptor blockers; ED, emergency department.

**FIGURE 2:**
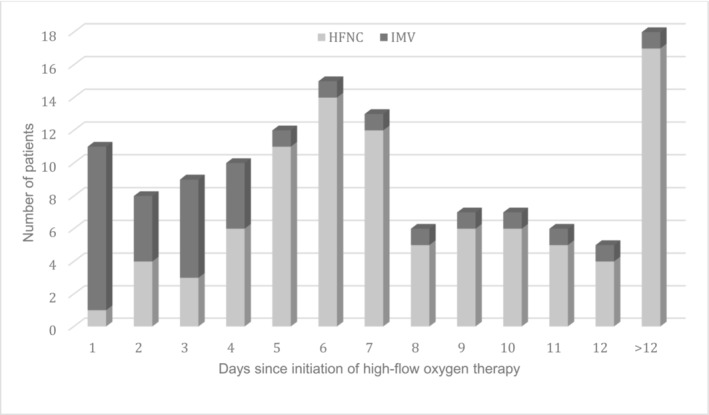
Patients intubated by duration of the high‐flow nasal cannula oxygen therapy.

The course of patients on HFNC is illustrated in Figure 2. Conversion to IMV was carried out a median of 3 days after starting HFNC. The median time on HFNC was 7 days^5^, ^6^, ^7^, ^8^, ^9^, ^10^, ^11^ in patients in whom the treatment was successful, compared to 3 days^1^, ^2^, ^3^, ^4^, ^5^ in those who eventually required IMV (*p* < 0.001).

## 4 DISCUSSION

The use of HFNC on the hospital ward enabled us to successfully treat a high percentage of patients (62%). If we consider only candidates for full escalation to IMV, the success rate was even higher (74%). To our knowledge, this is the first study carried out in Spain assessing HFNC outside ICU in a group of patients with severe AHRF secondary to SARS‐CoV‐2 infection.

The therapy was provided on a conventional hospital ward equipped to enable non‐invasive monitoring, demonstrating that this therapy is possible outside an ICU. Although this could be considered controversial, it is one of the strengths of this study, since it reflects the reality of the situation faced by hospitals during the pandemic. Based on our results, we can state that the strategy of using HFNC has enabled us to avoid ICU admission for more than half (53.9%) of patients.

A recent review of NIRS in patients with SARS‐CoV‐2 included 11 studies on HFNC in patients who were candidates for full treatment escalation, with a total of 1331 patients. Progression to IMV was observed in a median of 41% (IQR 29–52) of patients.^8^ Among our patients who were candidates for invasive procedures, the rate of treatment failure, defined as progression to IMV, was 25.9%, lower than in other studies. Panadero et al^9^ and Calligaro et al^10^ reported intubation rates of 52.5% and 43.2%, respectively, higher than in our study, despite our patients being older and having lower PaO2/FiO2 values. Delbove et al^11^ observed an intubation rate of 57% in a small number of patients (35), and while this is higher than rates observed in other studies, their patients were older (mean age of 73 years) though they did have a mean PaO2/FiO2 ratio >200. Other authors, based on 378 patients with acute respiratory failure secondary to SARS‐CoV‐2 in Mexico,^12^ found an intubation rate of 28.6%, slightly higher than in our study despite their patients being younger (mean age of 54.5 years).

Overall mortality rates vary considerably across studies, depending on the characteristics of patients studied (age, location of treatment, inclusion of patients with a ceiling of treatment). Patel et al^13^ reported a low mortality rate (14.4%) in 104 patients treated with HFNC on a hospital ward, but the mean age of patients included was 60.6 years, lower than that in our study. In the study carried out in Spain by Panadero,^9^ the overall mortality rate was 22.5%, based on 40 patients treated with HFNC in an intermediate respiratory care unit. In contrast, Calligaro et al^10^ reported a high mortality rate of 48% in 293 patients on HFNC, some of whom were not candidates for intubation. Considering only the patients who were eventually intubated, the mortality rate in our study was high (45%), similar to figures of 35%–45.4% reported elsewhere.^9^, ^11^, ^14^ Despite variability in the studies, they have all indicated that HFNC is useful in preventing progression to IMV. We did not select patients for inclusion; rather, we analysed the data on all patients in our hospital started on this type of therapy outside an ICU, which we considered to be a strength of our study.

A survival rate of 22% was found in the case of patients in whom HFNC was considered the ceiling of treatment (based on two studies with a total of 23 patients).^8^ According to these data, HFNC may also be a suitable treatment option for patients who are not candidates for IMV. In our series, the usefulness of HFNC was also observed in this group of patients, achieving a notably high rate of survival of 27.3% in the 44 patients for whom HFNC was considered the ceiling of treatment.

Despite these data, there is ongoing debate about the role of HFNC as well as the timing of intubation and the risk–benefit balance between patient self‐inflicted lung injury under spontaneous breathing and risks associated with intubation.^15^ HFNC was given for less time in patients who were subsequently intubated, as observed in other studies,^8^ indicating that failure occurs at an early stage. Nonetheless, there is currently no clear indication regarding the optimum timing of intubation. In 2015, Kang et al published a study on patients with severe hypoxaemic respiratory failure in which ICU mortality was higher in the group in which intubation was delayed for more than 48 h after HFNC initiation (66.7% vs 39.2% with earlier intubation, *p* = 0.001).^16^ In contrast, a retrospective analysis of 104 patients with moderate‐to‐severe AHRF secondary to SARS‐CoV‐2 pneumonia treated with HFNC found lower rates not only of IMV but also of mortality.^13^ More recently, Chavarria et al reported that 71.4% of patients with SARS‐CoV‐2 pneumonia and acute respiratory failure did not require IMV when treated with HFNC; this latter treatment improved respiratory parameters and seemed able to reduce the length of hospital and intensive care stays.^12^ Another multicentre study, carried out in Spanish and Andorran ICUs, concluded that the use of HFNC on ICU admission can increase the number of days without ventilation and reduce ICU stay, compared to results with early initiation of IMV.^17^ Lastly, a meta‐analysis including 8944 patients did not find differences in either mortality or duration of IMV between early intubation and intubation delayed for more than 24 h after ICU admission. Further, that analysis did not find significant differences in mortality between patients who did and did not receive HFNC before intubation.^18^

Investigating factors associated with HFNC failure, in our study, as in a previous study in patients with SARS‐CoV‐2 pneumonia^19^ and unlike what has been described for other conditions,^20^ PaO2/FiO2 at therapy initiation was not a reliable predictor of intubation. In the multivariate analysis, the only variables that remained significant were platelet count as a protective factor and creatinine levels and CALL score as risk factors. In line with this, various studies^21^, ^22^ have found SARS‐CoV‐2 severity and mortality to be related to lower platelet count. Further, our finding of CALL score at therapy initiation as a predictor of failure is consistent with the results of Chavarria et al^12^ Only 4.5% of our patients classified as low risk according to this scale (score <7) were intubated.

Our study has important limitations. The first is related to the small sample of patients included and that they come from a single centre. Secondly, the management of patients has varied considerably over the pandemic, with changes in pharmacological treatments used,^23^ vaccination status,^24^ levels of care/monitoring and levels of training and experience with the therapy among the staff involved. Similarly, there may have been differences in ICU admission as a function of levels of occupancy and overload in these units over time. All the variability that we underline as a limitation also has a positive side, namely, our study reflects the clinical activity as conducted, without patient selection and in a real‐world setting during these complicated months of the pandemic, with constantly changing conditions and challenges for healthcare management.

## 5 CONCLUSION

HFNC is a treatment with high success rate for patients with acute respiratory failure secondary to SARS‐CoV‐2 pneumonia, including patients for whom this therapy is considered a ceiling of treatment. This technique reduces the rates of intubation and mechanical ventilation, thereby avoiding numerous critical care admissions and leaving these beds for patients who do need intensive care. Finally, it is a therapy that can be used safely and effectively outside critical care settings, provided there are well‐trained staff and close monitoring. Multicentric studies comparing different respiratory support therapies are needed to confirm these results.

## AUTHOR CONTRIBUTIONS

**Sonia Castro:** Literature search; data collection; study design; analysis and interpreted the data; write the manuscript. **Sandra Pedrero:** Data collection. Study design. Analysis and interpreted the data. Review the manuscript. **Luis Alberto Ruiz:** Data collection; study design; analysis and interpreted the data; write the manuscript. **Leyre Serrano:** Data collection; study design; analysis and interpreted the data; write the manuscript. **Rafael Zalacain:** Data collection; study design; analysis and interpreted the data; write the manuscript. **Silvia Pérez‐Fernández:** Statistical analysis of data. **Milagros Iriberri:** Comment and revise the report. **Valentín Cabriada:** Literature search; data collection; study design; analysis and interpreted the data; write the manuscript. All authors read and approved the final manuscript.

## CONFLICT OF INTEREST STATEMENT

The authors have no conflicts of interest to declare.

## ETHICS STATEMENT

The study was approved by the Medical Research Ethics Committee of Euskadi (reference number: PI2020083) and was conducted in accordance with the principles of the Declaration of Helsinki on research involving humans.

## Data Availability

Research data are not shared.

## References

[1] Grasselli G , Zangrillo A , Zanella A , et al. Baseline characteristics and outcomes of 1591 patients infected with SARS‐CoV‐2 admitted to ICUs of the Lombardy region, Italy. JAMA. 2020;323(16):1574‐1581. doi:10.1001/jama.2020.5394 32250385 PMC7136855

[2] Casas‐Rojo JM , Antón‐Santos JM , Millán‐Núñez‐Cortés J , et al. Clinical characteristics of patients hospitalized with COVID‐19 in Spain: results from the SEMI‐COVID‐19 registry. Rev Clin Esp (Barc). 2020 Nov;220(8):480‐494. doi:10.1016/j.rce.2020.07.003 32762922 PMC7480740

[3] Ferrando C , Mellado‐Artigas R , Gea A , et al. (Eds). Patient characteristics, clinical course and factors associated to ICU mortality in critically ill patients infected with SARS‐CoV‐2 in Spain: a prospective, cohort, multicentre study. Rev Esp Anestesiol Reanim (Engl Ed). 2020;67(8):425‐437. doi:10.1016/j.redar.2020.07.003 32800622PMC7357496

[4] Gürün Kaya A , Öz M , Erol S , Çiftçi F , Çiledağ A , Kaya A . High flow nasal cannula in COVID‐19: a literature review. Tuberk Toraks. 2020 Jul;68(2):168‐174. English. doi:10.5578/tt.69807 32755117

[5] Nishimura M . High‐flow nasal cannula oxygen therapy in adults: physiological benefits, indication, clinical benefits, and adverse effects. Respir Care. 2016 Apr;61(4):529‐541. doi:10.4187/respcare.04577 27016353

[6] Rochwerg B , Granton D , Wang DX , et al. High flow nasal cannula compared with conventional oxygen therapy for acute hypoxemic respiratory failure: a systematic review and meta‐analysis. Intensive Care Med. 2019 May;45(5):563‐572. doi:10.1007/s00134-019-05590-5 30888444

[7] Ji D , Zhang D , Xu J , et al. Prediction for progression risk in patients with COVID‐19 pneumonia: the CALL score. Clin Infect Dis. 2020;71(6):1393‐1399. doi:10.1093/cid/ciaa414 32271369 PMC7184473

[8] Weerakkody S , Arina P , Glenister J , et al. Non‐invasive respiratory support in the management of acute COVID‐19 pneumonia: considerations for clinical practice and priorities for research. Lancet Respir Med. 2022 Feb;10(2):199‐213. doi:10.1016/S2213-2600(21)00414-8 34767767 PMC8577844

[9] Panadero C , Abad‐Fernández A , Rio‐Ramirez MT , et al. High‐flow nasal cannula for acute respiratory distress syndrome (ARDS) due to COVID‐19. Multidiscip Respir Med. 2020;15(1):693. doi:10.4081/mrm.2020.693 32983456 PMC7512942

[10] Calligaro GL , Lalla U , Audley G , et al. The utility of high‐flow nasal oxygen for severe COVID‐19 pneumonia in a resource‐constrained setting: a multi‐centre prospective observational study. EClinicalMedicine. 2020;28:100570. doi:10.1016/j.eclinm.2020.100570 33043285 PMC7536126

[11] Delbove A , Foubert A , Mateos F , Guy T , Gousseff M . High flow nasal cannula oxygenation in COVID‐19 related acute respiratory distress syndrome: a safe way to avoid endotracheal intubation? Ther Adv Respir Dis. 2021;15:1‐10. doi:10.1177/17534666211019555 PMC8170326 34057844

[12] Chavarria AP , Lezama ES , Navarro MG , et al. High‐flow nasal cannula therapy for hypoxemic respiratory failure in patients with COVID‐19. Ther Adv Infect Dis. 2021;3(8):1‐10. doi:10.1177/20499361211042959 PMC8419547 34497714

[13] Patel M , Gangemi A , Marron R , et al. Retrospective analysis of high flow nasal therapy in COVID‐19‐related moderate‐to‐severe hypoxaemic respiratory failure. BMJ Open Respir Res. 2020;7(1):e000650. doi:10.1136/bmjresp-2020-000650 PMC7451488 32847947

[14] Chandel A , Patolia S , Brown AW , et al. High‐flow nasal cannula therapy in COVID‐19: using the ROX index to predict success. Respir Care. 2021;66(6):909‐919. doi:10.4187/respcare.08631 33328179

[15] Brochard L , Slutsky A , Pesenti A . Mechanical ventilation to minimize progression of lung injury in acute respiratory failure. Am J Respir Crit Care Med. 2017;195(4):438‐442. doi:10.1164/rccm.201605-1081CP 27626833

[16] Kang BJ , Koh Y , Lim CM , et al. Failure of high‐flow nasal cannula therapy may delay intubation and increase mortality. Intensive Care Med. 2015;41(4):623‐632. doi:10.1007/s00134-015-3693-5 25691263

[17] Mellado‐Artigas R , Ferreyro BL , Angriman F , et al. Crit Care. 2021;25(1):58. doi:10.1186/s13054-021-03469-w 33573680 PMC7876530

[18] Papoutsi E , Giannakoulis VG , Xourgia E , Routsi C , Kotanidou A , Siempos II . Effect of timing of intubation on clinical outcomes of critically ill patients with COVID‐19: a systematic review and meta‐analysis of non‐randomized cohort studies. Crit Care. 2021;25(1):121. doi:10.1186/s13054-021-03540-6 33766109 PMC7993905

[19] Mellado‐Artigas R , Mujica LE , Ruiz ML , et al. COVID‐19 Spanish ICU network. Predictors of failure with high‐flow nasal oxygen therapy in COVID‐19 patients with acute respiratory failure: a multicenter observational study. J Intensive Care. 2021;9(1):23. doi:10.1186/s40560-021-00538-8 33673863 PMC7934982

[20] Frat JP , Thille AW , Mercat A , et al. High‐flow oxygen through nasal cannula in acute hypoxemic respiratory failure. N Engl J Med. 2015;372(23):2185‐2196. doi:10.1056/NEJMoa1503326 25981908

[21] Lippi G , Plebani M , Henry BM . Thrombocytopenia is associated with severe coronavirus disease 2019 (COVID‐19) infections: a meta‐analysis. Clin Chim Acta. 2020;506:145‐148. doi:10.1016/j.cca.2020.03.022 32178975 PMC7102663

[22] Yang X , Yang Q , Wang Y , et al. Thrombocytopenia and its association with mortality in patients with COVID‐19. J Thromb Haemost. 2020;18(6):1469‐1472. doi:10.1111/jth.14848 Epub 2020 May 4. PMID: 32302435 32302435 PMC9906135

[23] Mehraeen E , Najafi Z , Hayati B , et al. Current treatments and therapeutic options for COVID‐19 patients: a systematic review. Infect Disord Drug Targets. 2022;22(1):e260721194968. doi:10.2174/1871526521666210726150435 34313204

[24] Mehraeen E , Dadras O , Afsahi AM , et al. Vaccines for COVID‐19: a systematic review of feasibility and effectiveness. Infect Disord Drug Targets. 2022;22(2):e230921196758. doi:10.2174/1871526521666210923144837 34554905

